# New York Letter

**Published:** 1897-04

**Authors:** E. C. Davis

**Affiliations:** New York City


### CORRESPONDENCE.

### NEW YORK LETTER.

NEW YORK CITY, March 1, 1897.

There is a great amount of activity among the surgeons of this city at the present time. Daily notices present an array of very interesting operations, which the visitor may witness if fortunately situated. In speaking to one of the foremost surgeons about the apparent amount of work which he performed, he said, "I work hard for nine months in the year, and recreate three." On expressing surprise at the length of his recreative recess, he remarked, "Well, if we remain in the city throughout the entire year, our patrons conclude that we are not meeting with success, and several repetitions of this lead to a loss of patronage, for people always desire to place themselves under the care of the successful surgeon." Would that *we* of the South could educate our clientele to such ideas.

Perhaps it would be of interest to detail a few of the more interesting operations witnessed within the past few days at some of the hospitals.

At Roosevelt Hospital, a little more than a week ago, Dr. Chas. McBurney invited me to witness a nephrorrhaphy on a young girl for movable kidney. The usual incision was made, the kidney exposed, and its free movement during each respiratory act was plainly visible. After exposing the renal capsules, this was incised and sutured to the muscular tissue of the back with eleven catgut sutures. Dr. McBurney deprecates the practice of placing sutures deep into the cortical structure of the kidney, but after suturing the capsules does not completely close the incision; instead, he desires it largely to fill with granulations, producing a firm attachment of connective tissue. This requires a little longer time, but has been universally successful in his hands.

Another very interesting case which he showed was that of a man about fifty years of age, who for two years has been subject to recurring attacks of jaundice. At no time had he suffered severe pain as in biliary colic, he was greatly emaciated, appetite and digestion impaired, and never presented the characteristic evidences of jaundice.

A diagnosis of stone in common bile duct was made and that probably it was not impacted, but movable in a distended portion of the duct resembling the ball-valve of mechanics, and to which this name has often been given. The principle involved in the production of such a condition was described as follows: A stone becomes impacted in the common bile duct, and behind it the bile accumulates in sufficient quantity to thoroughly distend the duct and to make its lumen greater than that of the stone; then by a simple movement of the body the stone falls back, allows the bile to pass into the duodenum again, and the patient's condition improves. Then if the stone falls back and occludes the lumen again, a recurring attack of jaundice is produced, and the mechanical principle described above repeated. Sometimes, however, the stone becomes firmly impacted near the opening of the common bile duct into the duodenum, and when such is the condition, Dr. McBurney has devised an operation which he predicts will become the most generally adopted. At the present time it has only been performed a few times. It is the incision of duodenum, removal of stone through the dilated opening, and the suture of duodenal incision, which can be more securely sutured than the common bile duct.

In the case presented this could not be practiced, owing to distance from its opening into duodenum. Hence the duct, whose walls were very much thinned, was incised, the stone removed, the opening closed with small catgut sutures, and a large glass drainage tube wrapped with sterile gauze inserted.

The incision to reach this stone was a long perpendicular incision directly through the rectus abdominis muscle, careful attention being given to the hemorrhage before opening the peritoneum. One week later patient was shown in good condition; glass tube had been removed day before and a small rubber tube had replaced it. Leakage had occurred, but was at that time greatly diminished.

The removal by careful dissection of a large tumor of the thyroid was another very interesting operation performed by Dr. McBurney. This occurred in a young Swede, and had produced dyspnea; had become so large as to push the carotid several inches away from its normal location, and the cricoid cartilage rested under the angle of the jaw. This was removed by careful dissection from the body of the gland proper.

To me one of the most interesting cases of all occurred in a young man whose arm had been torn from his body three days before, leaving a jagged stump of soft tissue and a small portion of the humerus. The hemorrhage had not amounted to very much, owing to nature's method of clot formation following torsion, but at this time active sepsis was going on. The interesting feature was the extreme conservative plan adopted in the treatment of this case. After complete etherization, the parts were cleansed thoroughly with soap, water, and saline solution; then the infected area was trimmed off, leaving the bone exposed, but cleansed and healthy looking; then a few sutures placed approximating just a little the cut surfaces, the surface packed with gauze and allowed to granulate.

A week later the parts appeared healthy, the patient's temperature was normal, but the humerus still exposed. The reason assigned for this was that granulations would cover this bone, the small amount of bone left would preserve the rotundity of the shoulder and the usefulness of this side. Should, however, this not be covered, then it could easily be removed at a later day.

At the Woman's Hospital on Monday last Dr. Clement Cleveland performed a hysterectomy for ectopic pregnancy and infection and disease of both tubes and uterus. He first made an incision through the vagina, but finding the condition that of ectopic gestation with abundant adhesions, left this opening for drainage, and opened from above, uncovering tubes, ovaries, and uterus.

On Wednesday saw Dr. H. T. Hanks perform a hysterectomy for uterine fibroid by morcellation.

